# A new balloon dissector for totally extraperitoeneal hernia repair

**DOI:** 10.4103/0972-9941.51318

**Published:** 2009

**Authors:** Sunil Kumar

**Affiliations:** Department of Surgery, Guru Teg Bahadur Hospital and University College of Medical Sciences, Dilshad Garden, Delhi-110 095, India

**Keywords:** Balloon dissector, retroperitoneoscopy, totally extraperitoneal

## Abstract

**BACKGROUND:**

Balloon dissectors (BD) find their use in totally extraperitoneal (TEP) and retroperitoneoscopic procedures. Commercial BD is prohibitively expensive. The author uses an indigenously assembled BD and describes the same.

**MATERIAL AND METHODS:**

The author assembles the BD by tying glove-fingers on an NG tube and then tying this assembly in the concavity of a Kelly's clamp, premounted with peanut gauze (KC-BD).

**RESULTS:**

The author has used it in the last 75 cases of TEP. A large working space is created, without any iatrogenic injuries or balloon rupture. This cheap indigenous BD can be assembled easily and in no time at all.

**CONCLUSIONS:**

KC-BD offers several advantages because of its unique design. It is effective, totally nontraumatic, inexpensive, and easy to assemble.

## INTRODUCTION

To perform totally extraperitoneal (TEP) repair of hernia and retroperitoneoscopic procedures, it is essential to create an initial working space for placement of, first, the camera port, and then, the ports for accessory instruments. Although experienced surgeons create this initial working space under vision, the beginners find it easy to do so by using a balloon dissector (BD).[1] Commercially available BDs are nontraumatic, but expensive. Therefore, indigenously designed BDs are popular.[2,3] The simplest indigenously designed BD, assembled by tying ‘glove-fingers’ over a 10F red-rubber catheter,[2] is difficult to insert as the catheter kinks. The author has designed his own indigenous BD (referred to as KC-BD, since it is assembled by using a Kelly's clamp). The author is very satisfied using KC-BD when performing TEP and is reporting the same.

## ASSEMBLING KC-BD

The following presterilized materials are needed:

1. One 12-14F nasogastric (NG) or any other tube, stiff enough to resist compression by silk threads being tied over it. The terminal part of the NG tube, containing multiple holes, is discarded.
2. Two ‘glove-fingers’ of same size, cut from surgical gloves.
3. Two strong 10-inch-long silk threads.
4. One artery clamp.
5. One peanut gauze mounted Kelly's clamp.

Basically, only three steps are essential to assemble this indigenous BD:

Step 1. Tie the mouths of both glove-fingers over an NG tube using silk sutures, keeping the suture ends long, [Figure 1a].

**Figure 1a:**
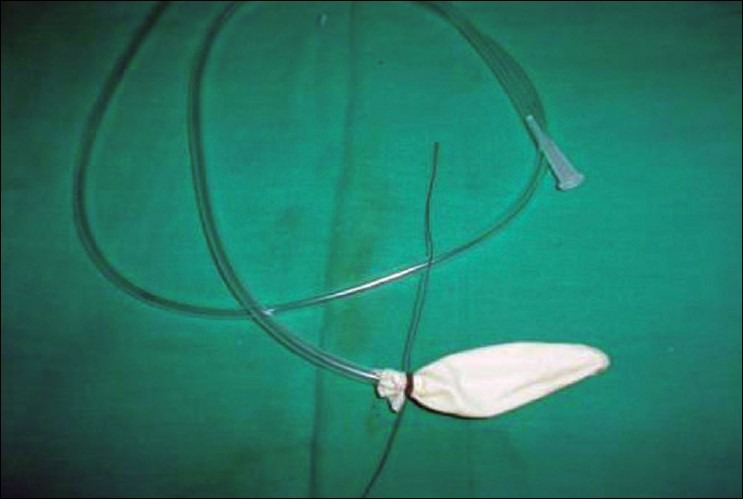
Mouth of glove-fingers tied over NG tube; threads kept long

Step 2. Holding the tip of the glove-finger (with an artery clamp), tie a second silk suture at its tip, avoiding the inside of the NG tube; suture-ends are kept long, again, [Figure 1b].

**Figure 1b:**
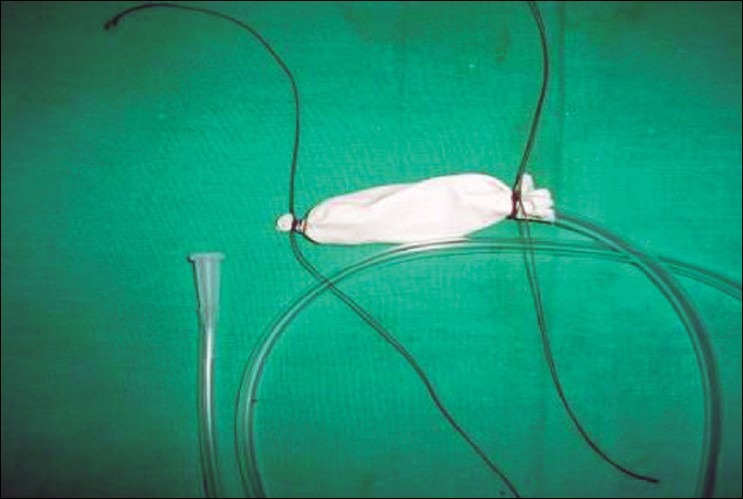
Tip of glove-fingers tied excluding NG tube; threads kept long

Step 3. Spread this assembly evenly along the concavity of the Kelly's clamp, so that the NG tube runs backward along the blade of the Kelly's clamp. First tie the distal thread over the Kelly's clamp, just below the peanut gauze [Figure 1c], and then tie the proximal thread over the Kelly's clamp, as far back as possible, keeping the finger gloves spread evenly, [Figure 1d]. Trim threads short.

**Figure 1c:**
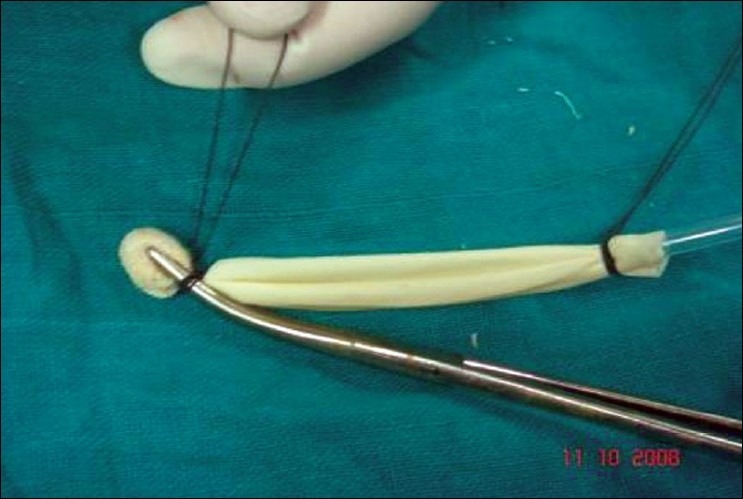
Glove-finger and NG tube assembly placed in concavity of Kelly's clamp, preloaded with peanut gauze; distal thread tied over the Kelly's clamp, just below the peanut gauze

**Figure 1d:**
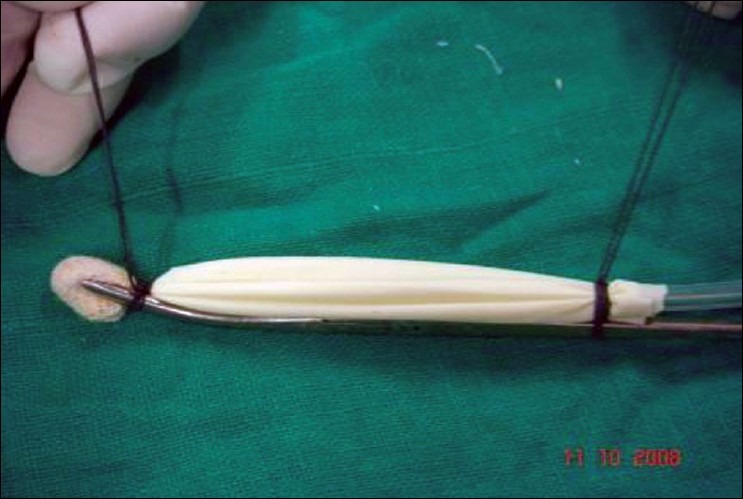
Keeping glove-fingers straight, proximal thread tied over the Kelly's clamp

The new indigenous BD is ready for use [Figure 1e].

**Figure 1e:**
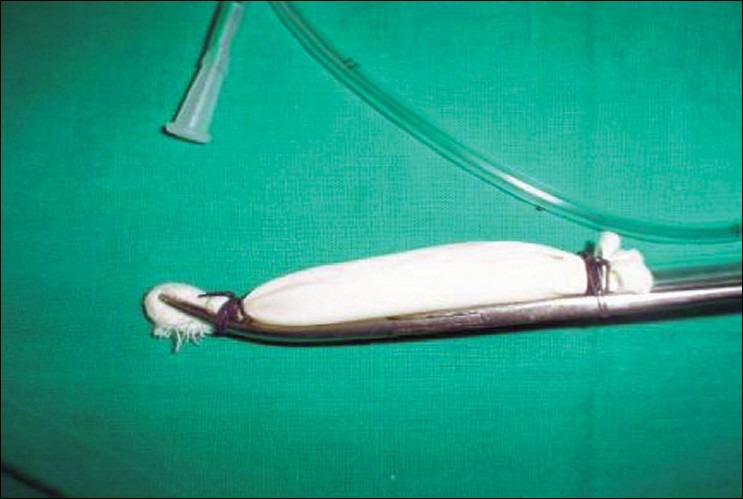
KC-BD is ready

## TECHNIQUE OF INSERTING KC-BD (IN TEP)

After incising the anterior rectus sheath horizontally, retract and hold the rectus muscle laterally. Grasp the Kelly's clamp in the palm, with the index finger and thumb providing appropriate support. Keeping the concavity of the Kelly's clamp facing the surgeon's belly, first negotiate the tip of the KC-BD (which has a peanut gauze) behind the rectus muscle. Thereafter, advance the KC-BD gradually and turn it up so that the concavity of the Kelly's clamp faces the rectus muscle. Normally, the tip of the KC-BD can easily be advanced up to the pubic bones. The KC-BD can now be inflated using (up to) 150 cc of air, by attaching a pump to the open end of the NG tube [Figure 1f]. The balloon is kept inflated for 60 seconds, then deflated and gradually withdrawn. During withdrawal, the movements are reversed for a safe exit. This leaves a large bloodless cavity behind the rectus muscle in which a camera and accessory ports can be inserted, for subsequent operative steps.

**Figure 1f:**
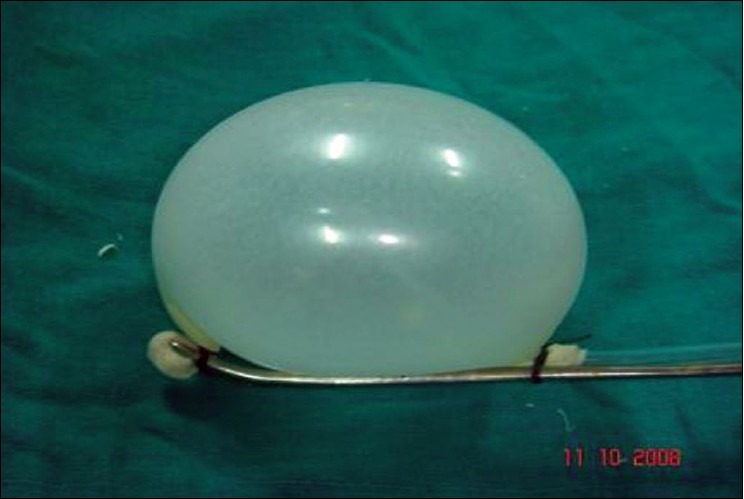
KC-BD is tested; note the perfect hemishperical shape of the balloon

## DISCUSSION

The peanut gauze at the tip of KC-BD serves two purposes: (i) it bluntly dissects the tunnel behind the rectus muscle, to accommodate the balloon portion of the BD, and (ii) it imparts a certain degree of nontraumatic nature to this instrument when executing the first function. The short length of the KC-BD also facilitates perfect control at the tip of this BD and contributes to its nontraumatic property. The balloon of this BD inflates uniformly in a hemispherical form and is not prone to rupture. KC-BD deserves use on a larger scale.
